# Mechanisms of Schwann cell plasticity involved in peripheral nerve repair after injury

**DOI:** 10.1007/s00018-020-03516-9

**Published:** 2020-04-10

**Authors:** Gianluigi Nocera, Claire Jacob

**Affiliations:** Faculty of Biology, Institute of Developmental Biology and Neurobiology, Johannes Gutenberg University, Mainz, Germany

**Keywords:** Schwann cell, Plasticity, Reprogramming, Chromatin remodeling enzymes, Transcription factors, Signaling pathways, Nerve injury and repair, Axonal regeneration, Remyelination

## Abstract

The great plasticity of Schwann cells (SCs), the myelinating glia of the peripheral nervous system (PNS), is a critical feature in the context of peripheral nerve regeneration following traumatic injuries and peripheral neuropathies. After a nerve damage, SCs are rapidly activated by injury-induced signals and respond by entering the repair program. During the repair program, SCs undergo dynamic cell reprogramming and morphogenic changes aimed at promoting nerve regeneration and functional recovery. SCs convert into a repair phenotype, activate negative regulators of myelination and demyelinate the damaged nerve. Moreover, they express many genes typical of their immature state as well as numerous de-novo genes. These genes modulate and drive the regeneration process by promoting neuronal survival, damaged axon disintegration, myelin clearance, axonal regrowth and guidance to their former target, and by finally remyelinating the regenerated axon. Many signaling pathways, transcriptional regulators and epigenetic mechanisms regulate these events. In this review, we discuss the main steps of the repair program with a particular focus on the molecular mechanisms that regulate SC plasticity following peripheral nerve injury.

## Schwann cells and peripheral nerve injuries

**Fig. 1:**
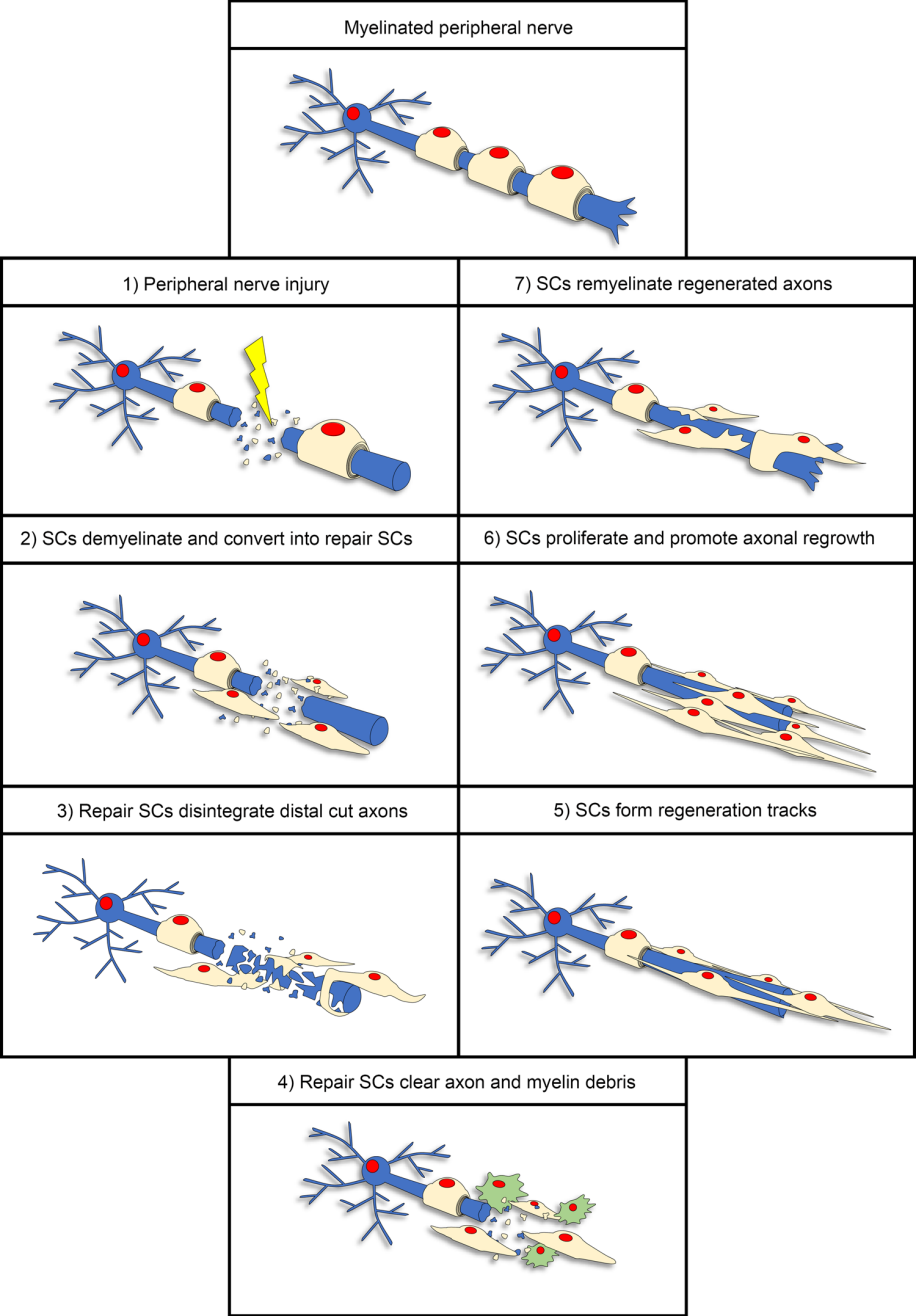
Repair program in the PNS. Illustration of the main steps of the repair program orchestrated by SCs after peripheral nerve injury. Each step shows a schematic representation of a single neuron (blue) interacting with SCs (light yellow) within an adult peripheral nerve undergoing a traumatic lesion. In step 4, macrophages (green cells) help SCs to clear axon and myelin debris

Axonal repair in the central nervous system (CNS) is extremely limited after injury. In contrast, the PNS exhibits a high regenerative capacity. This ability is to a large extent due to the remarkable plasticity of SCs. During development, myelinating SCs form a one-to-one relationship with large caliber axons and wrap them in a myelin sheath, while non-myelinating SCs, also called Remak SCs, surround multiple small caliber axons without producing myelin. Upon axon injury, myelinating and non-myelinating SCs undergo extensive reprogramming that promotes and guides axonal repair. SCs lose contact with and demyelinate the distal stump axon and convert into a repair phenotype. This phenotypic transformation involves the downregulation of several pro-myelinating genes. Repair SCs are characterized by a specific profile which enables the regeneration process. SC reprogramming involves the upregulation of several genes and the activation of multiple transcriptional mechanisms [1–3]. Among the main players, c-Jun, mitogen-activated protein kinase (MAPK) pathways, Sonic Hedgehog (Shh) and chromatin modifications control and regulate the repair program. In the injured site, repair SCs participate in the disintegration and removal of damaged axons during the process of Wallerian degeneration and assist myelin debris clearance to create a regrowth favorable environment. Myelin debris clearance is achieved by the digestion of both intrinsic and extrinsic myelin fragments by means of myelinophagy and phagocytosis and the recruitment and activation of invading macrophages [4, 5]. Afterwards, SCs secrete trophic factors to support survival of damaged neurons and promote axon regrowth [1, 6]. Repair and Remak SCs also extend long parallel processes and align in tracts called bands of Büngner to guide the regrowing axon back to innervate its former target [3, 7]. Finally, SCs proliferate, upregulate pro-myelinating genes, re-differentiate into myelinating SCs and remyelinate the regenerated axon. An overview of the repair program is illustrated in Fig. 1. This repair machinery requires a dynamic and orchestrated regulation of SC plasticity and reprogramming following axon injury. Although the underlying molecular mechanisms remain still partially understood, several research groups have significantly contributed to our current understanding of how the key steps involved in the PNS repair program are controlled, which we will discuss in the next chapters of this review.

## Nerve injury and methods in regeneration research

The severity of peripheral nerve injuries is classified depending on whether demyelination occurs and on the extent of axonal and connective tissue damage [8]. The mildest form called neurapraxia is characterized by local demyelination without axon or connective tissue lesion. Axonal lesion in addition to demyelination but with preserved connective tissue is a more severe injury called axonotmesis, while in the most severe form of injury, which is called neurotmesis, axons and the connective tissue are fully transected [9]. A more detailed classification with five different severity degrees followed Seddon’s classification [10]. SC plasticity and regeneration potential have been extensively studied both in vivo and in vitro. In vivo studies involved the use of wild type and transgenic animals. Among the methods used to study regeneration, nerve transection and crush injury models are the most commonly employed. The crush injury model offers some particular advantages. It is typically performed through an acute traumatic compression of the nerve and it interrupts all the axons but preserves the SC basal lamina. This allows for an optimal regeneration and to investigate the SC regeneration potential. Rats and mice are often used in research for sciatic nerve lesions to model human PNS lesions [11, 12]. In vitro studies mainly involve the use of cell lines, primary or organotypic ex vivo cell cultures. These models present great ethical advantages and they allow to investigate signalling pathways specifically induced by different molecules and drugs on SCs and/or neurons [13, 14]. Although they are unable to fully predict what happens at the whole organ level, primary SC/neuron cocultures have been useful PNS models to investigate the molecular mechanisms involved in axonal regrowth fostered by SCs and remyelination. Moreover, microfluidic devices, which allow the compartmentalization of neuronal cell bodies, axons and myelinating cells, have demonstrated to be useful in in vitro research [15, 16].

## Schwann cell reprogramming

**Fig. 2:**
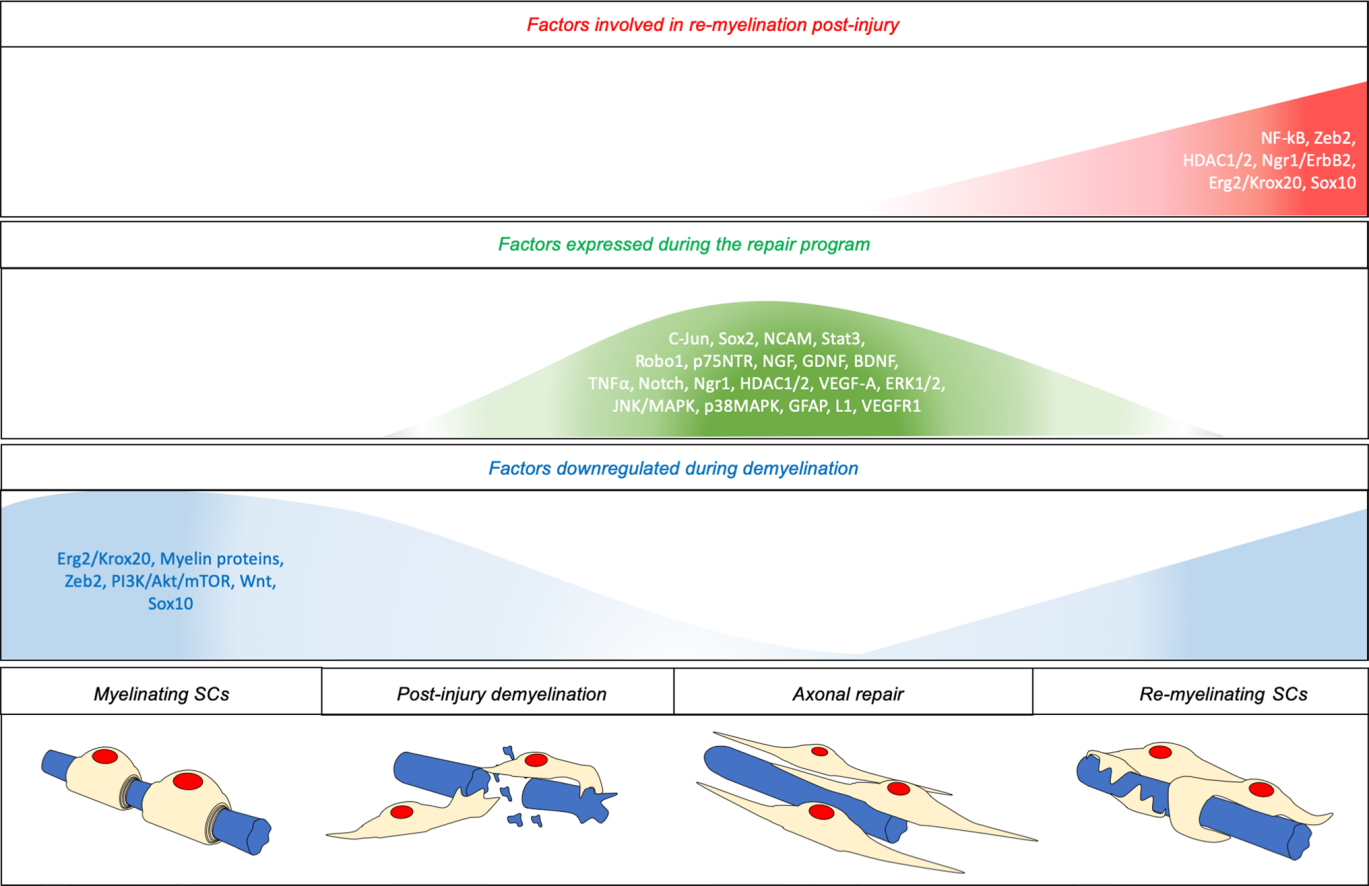
Summary of the main factors regulated after peripheral nerve injury. Overview of factors downregulated during demyelination (blue area), factors expressed during the repair program (green area), and factors involved in re-myelination post-injury (red area). Uphill areas indicate a high expression, whereas downhill areas indicate a low expression. Regulation of factors is illustrated throughout the different steps of the regeneration process

SC reprogramming following peripheral nerve injury can be divided in two main partially overlapping processes: SC demyelination and conversion or transdifferentiation into repair SCs [5, 17]. The demyelination process is characterized by the repression of pro-myelinating genes such as *Early growth response 2* (*Erg2 or Krox20*) and of myelin genes including *Myelin basic protein (Mbp), Myelin protein zero *(*Mpz or P0*)*, Peripheral myelin protein 22 *(*Pmp22*) and *Myelin associated glycoprotein* (*Mag*). Moreover, genes typically expressed during development including genes coding for L1, neural cell adhesion molecule (NCAM), p75 neurotrophin receptor (p75NTR) and glial fibrillary acidic protein (GFAP), are re-expressed or upregulated [17, 18]. However, repair SCs differ from immature SCs during development by several aspects. Indeed, they are characterized by de-novo expression of genes that include *Olig1* and *Shh* and by the upregulation of many proteins involved in the regeneration process. Among those, (1) c-Jun, the main driver of the SC-dependent repair program, glial cell-derived neurotrophic factor (GDNF), brain-derived neurotrophic factor (BDNF), neurotrophin-3 (NT3), artemin, nerve growth factor (NGF), vascular endothelial growth factor (VEGF), and VEGF receptor 1 support the survival of injured neurons and promote the regrowth of proximal axons [1, 16, 18–20]; (2) leukemia inhibitory factor (LIF), interleukin-1α (IL-1α) and -1β (IL-1β), tumor necrosis factor-α (TNF-α) and monocyte chemotactic protein 1 (MCP-1) initiate the immune response, promote macrophage invasion and activation, blood vessel formation and myelin breakdown [1, 21–24]; (3) c-Jun, SRY-box 2 (Sox2) and neuregulin 1 (NRG1) are involved in SC morphological changes and axoglial interactions, in the formation of a nerve bridge in case of nerve transection and of the regeneration tracks along which axons regrow; (4) zinc finger E-box-binding homeobox 2 (Zeb2), nuclear factor-kappa B (NF-kB) and histone deacetylases 1 and 2 (HDAC1/2) are involved in the remyelination of the regenerated axon [25–29]. A summary of the main factors involved in the regeneration process is illustrated in Fig. 2, while a more detailed description of the main factors and signaling pathways with the most recent findings is discussed below.

## Epigenetic regulation and other chromatin-remodeling enzyme functions

The complexity of the repair program requires a strict orchestration of gene expression and signalling pathways. Among the many levels of regulation, epigenetic regulation and chromatin-remodeling enzymes have been related to many aspects of SC development, maintenance and plasticity after peripheral nerve injury [2]. In SCs of uninjured nerves, Polycomb repressive complex 2 (PCR2) adds trimethyl marks to histone H3 lysine 27 (H3K27me3) on promoter regions of several genes to repress the expression of these genes and PRC2 inactivation results in the induction of genes that are normally upregulated following peripheral nerve injury [30]. Upon injury, H3K27 demethylation together with H3K4 methylation at promoter regions and H3K27 acetylation at enhancer regions promote the activation of injury-induced genes expressed during the repair program such as *Shh* and *Gdnf* [2, 30–32]. Recently, much research has focused on the functions of HDACs. By deacetylating and controlling the activity of non-histone targets including transcription factors, these enzymes have been shown to play key roles in the regulation of SC behavior following peripheral nerve lesion. HDAC1 and HDAC2, which belong to class 1 HDACs, have crucial functions in SC development including myelination and in PNS maintenance during adulthood [1, 24, 33]. Moreover, they are strongly upregulated following axonal injury [29]. Recent studies revealed that by interacting with Sox10, HDAC2 recruits the H3K9 demethylases KDM3A and JMJD2C to form a complex that de-represses and activates *Oct6* and *Krox20* genes during the repair program. In turn, Oct6, which is upregulated too early after lesion, negatively regulates c-Jun expression and thereby delays the conversion of SCs into repair SCs. Genetic inactivation of HDAC1/2 prevents Oct6 and Krox20 upregulation after lesion, which results in faster axonal regrowth but impairs the remyelination process. Interestingly, short-term treatment early after lesion with Mocetinostat, a pharmacological inhibitor of HDAC1/2, accelerates the regeneration process without impairing the remyelination process [29]. These findings are of particular interest for the development of potential treatments to improve peripheral nerve regeneration when lesions have led to large gaps between axons and their targets. Indeed, functional recovery of peripheral nerves after lesion is critically dependent on the speed of axon reconnection to their former target [34]. HDAC3, another class 1 HDAC, has been shown instead to limit myelination [35, 36]. Indeed, HDAC3 inactivation by pharmacological inhibition or by conditional deletion in SCs enhances myelin growth after peripheral nerve injury or during PNS maintenance in adulthood [35, 36], which could thus be potentially interesting in human medicine to improve PNS remyelination after injury and prevent demyelination during aging or in the context of peripheral neuropathies. He et al. [35] and Rosenberg et al. [36] propose, however, two very different mechanisms of action for HDAC3: while He et al. [35] claim that HDAC3 antagonizes the neuregulin-PI3K-AKT signaling pathway and coordinates with p300 histone acetyltransferase to repress the promyelinating program by epigenetic silencing and to activate genes that inhibit SC myelination, Rosenberg et al. [36] found that HDAC3 allows to switch off the HDAC1/2-dependent biogenic myelination program to enter the homeostatic myelination program in adult peripheral nerves. Clarification of this process is, therefore, needed. Epigenetic regulation has been also shown to have critical functions in ensuring a correct functioning of SCs during the repair program. Indeed, during demyelination, SCs re-enter the cell cycle and proliferate. To prevent uncontrolled proliferation that could lead to tumor formation, SCs upregulate JMJD3 after a nerve lesion, which demethylates H3K27 at promoter regions and de-represses the tumor-suppressor p19Arf and p16Ink4a [37].

## Axon and myelin clearance

To be efficient, the regenerative process requires the generation of a favorable environment that fosters axonal repair. Rapidly after injury, SCs promote the disintegration of distal cut axons [16] and their clearance, together with invading macrophages [38–40]. The presence of persistent axon fragments inhibits the regeneration of axon branches [21] and leads to delayed axonal regrowth [16, 41, 42]. We recently showed that after a peripheral nerve lesion, SCs form constricting actomyosin spheres along unfragmented distal cut axons to accelerate their disintegration [16]. Interestingly, this mechanism is triggered by distal cut axons that upregulate the VEGFR1 agonist PlGF by injury-induced local translation, resulting in the activation of a VEGFR1/Pak1/F-actin axis in SCs [16]. Moreover, after injury myelin in the distal injured axon site breaks down into small intracellular and extracellular fragments and debris. Myelin debris act as an inhibitor of axon regeneration by creating a non-permissive environment which impairs axonal regrowth. However, PNS myelin is less inhibitory than CNS myelin [43] and numerous mechanisms in the PNS contribute to myelin digestion after nerve injury. In the first phase after nerve damage, intrinsic myelin is digested by SCs through a type of selective macro-autophagy called myelinophagy [4]. During this process, intracellular myelin debris are sequestered by a double membrane phagophore, which matures into an autophagosome and finally fuses with a lysosome triggering the degradation of the autophagic cargo. Myelinophagy appears to be essential during the early stages after injury, as genetic and/or pharmacological inhibition of autophagy inhibits myelin protein and lipid breakdown in injured nerves [4]. During the second phase post injury, SCs and invading macrophages collaborate to clear extrinsic myelin. SCs engulf and digest myelin debris by receptor-mediated phagocytosis. In particular, Axl and Mertk, two well-characterized receptors belonging to the TAM family of phagocytic receptors, appear to be critical for myelin digestion, as shown by impaired myelin degradation in SCs lacking these receptors [44]. SCs participate to macrophage recruitment to the injured site. Macrophages play a critical role in peripheral nerve injury. Besides contributing to myelin clearance, they participate in the inflammatory response, foster axon debris removal and regulate the injured site microenvironment, which allows for efficient regeneration. During the peak phase of myelin clearance after injury, repair SCs express high levels of several growth factors and chemoattractant cytokines including MCP-1, GDNF, interleukin-6 (IL-6) and LIF. These factors act by promoting the recruitment and the induction of both pro- and anti-inflammatory macrophages (M1- and M2-macrophages) [45–48].

## Signaling pathways and transcription factors

### NRG1 and its dual role in regeneration

It has been already established that the NRG1/ErbB signaling pathway is critically involved in axoglial communication regulating myelination and the thickness of the myelin sheath during PNS development, and in SC proliferation, survival and migration [49–57]. During adulthood, different NRG1 isoforms play different roles. Transmembrane NRG1 isoforms are expressed by myelinated axons, while soluble NRG1 isoforms are expressed by Schwann cells immediately after injury. NRG1/ErbB signaling is highly regulated following nerve injury [58–61]. Indeed, NRG1 and ErbB2/3 receptor levels significantly increase in distal nerve stump SCs [59]. In contrast, the expression of NRG1 by peripheral neurons initially decreases and then increases as axons re-innervate their targets [59, 62]. NRG1 type III expression in neurons appears to be required for timely repair and remyelination. Indeed, transgenic animals in which axons lack NRG1 type III show slower regeneration after nerve crush and display a significant transient impairment in remyelination following injury [27, 63, 64]. NRG1/ErbB signaling is regulated by beta-site amyloid precursor protein cleaving enzyme 1 (BACE1), which cleaves and activates NRG1 type III and is necessary for myelination during development [65, 66]. Moreover, mice lacking BACE1 display delayed remyelination, reduced myelin thickness in remyelinated axons and a general reduction in the total number of remyelinated axons. However, these mutants surprisingly show faster axon and myelin debris clearance and axon regeneration and reinnervation of their target [67, 68]. In addition to axonal NRG1 type III, NRG1 type I expressed by SCs upon injury is necessary for remyelination after lesion [69]. Based on these evidences, a number of studies focused on the application of exogenous NRG1 to increase NRG1 levels and improve axonal regeneration, remyelination and functional recovery following peripheral nerve injury [27, 69–72]. Interestingly, rats treated with an ErbB2 receptor inhibitor display a reduction of demyelination after transection [73], which suggests that the NRG1/ErbB signaling may also have a function in myelin breakdown. The pathway downstream NRG1/ErbB signaling is still not fully characterized. While some studies propose that the activation of ErbB2/3 in SCs by NRG1 promotes ERK activation and induces SC demyelination and conversion into repair cells or remyelination [69, 74], others suggest that NRG1 triggers Rac/JNK activation [13, 75]. It is important to point out that the apparently opposite functions of the NRG1/ErbB signaling seem to be due to different stimulation levels of the pathway and that an uncontrolled or inappropriate induction of the NRG1/ErbB signaling may be harmful as it could lead to demyelinating neuropathy as well as neoplastic conditions [74, 76–78].

### c-Jun

c-Jun is a key transcription factor involved in SC reprogramming and response to peripheral nerve injury. Its expression is constitutively low, both during development and in adult nerves, and it does not appear to be crucial in adult uninjured nerves, as transgenic animals carrying conditional deletion of c-Jun (c-Jun cKO) in SCs appear normal. By contrast, *c-Jun* gene expression rapidly increases following nerve injury and in some peripheral neuropathies and is critical during axonal regeneration. Indeed, c-Jun cKO mice exhibit a delayed demyelination, decreased neuronal survival and limited regeneration capacity [3, 79–83]. In injured nerves, c-Jun affects the expression of hundreds of genes and regulates several aspects of the regeneration process [3, 19]. Several lines of evidence support the idea that c-Jun is directly involved in and drives SC demyelination and transdifferentiation into a repair phenotype. As demyelination requires the downregulation of several pro-myelinating genes, c-Jun antagonistic expression with Krox20 led to identify this factor as the main negative regulator of myelination driving demyelination. Indeed, enforced c-Jun expression is enough to inhibit myelination in neuron/SC cocultures [3, 80, 81]. c-Jun functions have been also correlated with myelin breakdown during the process of myelin clearance, as shown by a significant reduction in myelin debris degradation following nerve transection in transgenic mice carrying a conditional deletion of c-Jun in SCs. Although this impairment is correlated with a deficit in the myelinophagic pathway, it is unclear whether this effect is related to myelinophagy only or to a defect in both myelinophagy and myelin phagocytosis. Myelin breakdown impairment could also be related to a defect in macrophage activation, as in these mutants, macrophages contain an increased amount of lipid droplets as compared to controls. In addition, evidence supports the idea that c-Jun also acts as an intrinsic determinant of SC morphology and controls the structure of the regeneration tracks that guide growing axons back to re-innervate their former targets [3, 4, 84]. In conclusion, c-Jun appears to be a global regulator of the SC-dependent repair program. Several signalling pathways have been proposed to be upstream of and to regulate c-Jun expression. Among these, the MAPK pathways extracellular signal-regulated protein kinases (ERK)1/2, c-Jun N terminal kinase (JNK) and p38 are plausible candidates, all of them being activated following nerve injury [85–87].

### MAPK functions in nerve repair

Many MAPKs have been involved in SC plasticity and axonal regeneration. Their role in the repair process is quite complex and has been sometimes controversial with different studies displaying conflicting results. The MAPK/ERK, JNK and p38MAPK signaling pathways are regulated after nerve injury. MAPK/ERK is involved in many physiological processes including metabolism, survival, apoptosis, proliferation and cell differentiation [88, 89]. In both PNS and CNS, the ERK1/2 pathway appears critical for myelination and myelin maintenance [90–92]. However, SCs respond to peripheral nerve injury by a strong activation of the ERK pathway [93] and in the absence of nerve injury, sustained activation of the ERK pathway in SCs is sufficient to drive demyelination and induce an inflammatory response [86]. The latter findings would be compatible with the idea that c-Jun is a downstream target of ERK signaling [86], although this hypothesis is quite controversial. Indeed, while some authors showed that in vivo ERK overactivation induces c-Jun upregulation and ERK inhibition negatively affects demyelination and c-Jun expression in myelinating co-cultures [86, 94], others reported that c-Jun upregulation and SC demyelination are JNK or p38MAPK-dependent [13, 95, 96]. Moreover, Cervellini et al. [97] reported that sustained activation of the ERK pathway in injured nerves resulted in delayed repair and functional recovery. Indeed, this study shows that early after injury, myelin clearance is faster in mutant mice expressing high and sustained levels of MAPK/ERK in SCs, but 4 weeks following injury, mutant nerves display reduced myelin compaction, reduced number of Cajal bands, decreased internodal length and fewer regenerated axons [97]. Other studies showed instead pro-myelinating effects of ERK activation and that ERK ablation results in inhibition of SC differentiation and myelination in vivo [92, 98, 99]. These apparent contradictory functions suggest that MAPK/ERK acts over multiple pathways regulating different processes during development and the repair program. More work is, however, required to address this hypothesis.

Another member of the MAPK family, p38MAPK, has also been shown to play a critical role in SC plasticity following injury. It has been suggested that by mediating laminin signaling, p38MAPK regulates SC elongation and alignment along axons, which is required for myelination [100]. In the CNS, p38MAPK has also been shown to be essential during development for oligodendrocyte progenitor proliferation, differentiation and myelination [101–104]. However, a different role has been proposed for p38MAK during the repair program. Following peripheral nerve injury, p38MAPK appears important to initiate the injury response. Early after injury, p38MAPK activity is rapidly increased [85] and in vivo inactivation blocks SC demyelination and conversion into repair cells in the distal nerve [96]. Indeed, p38MAPK promotes SC demyelination and conversion into repair SCs by downregulating myelin proteins and upregulating c-Jun expression [96]. This study also suggests that p38MAPK mediates myelin breakdown and inactivation promotes myelination in co-cultures. Moreover, by employing a *p38a* mutant mouse line, which displays a limited gene loss-of-function, Kato et al. [105] reported that the p38MAPK signaling is also important to initiate the inflammatory response after lesion. Indeed, p38a insufficiency results in inflammatory disorder and delay of histological and functional nerve recovery [105]. Therefore, several evidences support the role of p38 MAPK as a negative regulator of SC myelination, driving the initial response to injury.

Such as the ERK1/2 and the p38MAPK pathways, the JNK pathway is also rapidly activated after nerve injury [81] and has also been involved in several important functions controlling SC plasticity. In in vitro experiments, JNK activation results in the inhibition of myelin gene expression, SC demyelination, proliferation, upregulation and phosphorylation of c-Jun [75, 80, 81, 95, 106–108]. Indeed, c-Jun is known to be a major phosphorylation target of JNK, although the requirement of phospho-c-Jun after nerve injury has not yet been clarified [13, 95]. However, in the distal stump of injured nerves, JNK inhibition decelerates myelin fragmentation [109], which suggests a function of c-Jun phosphorylation in accelerating demyelination. Consistent with this hypothesis, the JNK1/c-Jun pathway can stimulate autophagy in several cell types [110] and pharmacological or genetic inactivation reduces SC autophagic flux [4]. Therefore, the JNK pathway appears to regulate several aspects of the SC repair program by promoting the activation  of c-Jun and expression of others factors associated with the repair program, such as GDNF and P75NTR [13].

### Sox2 and nerve bridge tissue formation

Regeneration and reinnervation are often successful after nerve crush injuries, as the basal lamina surrounding the axons is often preserved. Regeneration after more severe injuries such as nerve transection is, however, generally less efficient. Indeed, upon cut, a gap forms between the two nerve stumps [111]. A proper regeneration and reinnervation requires the formation of a bridge connecting the two extremities. Sox2 has been identified as another negative regulator of myelination and remyelination induced in repair cells after injury [112, 113], and has been shown to play an important role in tissue bridge formation between the proximal and distal nerve stumps by mediating the ephrin-B/EphB2 signaling between fibroblasts and SCs and the relocalization of N-cadherin between SCs [26]. Ephrin/Eph is an important mediator of bidirectional signaling between axons and glial cells in the nervous system and has already been shown to be involved in multiple processes such as scar formation, axon guidance, axonal regeneration, and myelination [114]. Nerve damage repair SCs gather at both nerve stumps, where they come into direct contact with fibroblasts, which accumulate at the wound site. Ephrin-B/EphB2 mediate the cell sorting and orchestrate the collective migration of SCs to form multicellular cords that guide and direct the axons across the injury site. Indeed, it has been shown that both pharmacological inhibition and genetical ablation of EphB2 resulted in significantly shorter and less organized regrowing axons as compared to untreated and wild type animals [26]. Moreover, Dun et al. [115], showed that by regulating the Slit3/Robo1 pathway, Sox2 is also critical for SC migration in the nerve bridge and axon pathfinding after nerve injury. Indeed, this study shows how Sox2 loss of function leads to ectopic SC migration and to the inability to form proper SC cords connecting the nerves stumps [115]. It has previously been shown that Slit-Robo interaction is crucial for axon guidance and serves as a repulsive signaling to control axon pathfinding and neuronal migration during nervous system development [116]. In their study, Dun et al. showed that Sox2 regulates Robo1 receptor expression in migrating SCs and that macrophages in the outermost layer of the nerve bridge secrete high levels of its ligand Slit3. Further gene ablation experiments confirm that Slit3/Robo1 repulsive signal is crucial for SC migration trajectory and correct nerve bridge formation following nerve transection [115]. Taken together these results suggest that the coordinated action of different cell types and the combined function of multiple axon guidance molecules is necessary for a correct nerve bridge tissue formation and precise axon targeting in the nerve bridge. Indeed, it has also been shown that a crosstalk between macrophages and endothelial cells is both necessary and sufficient for SCs to find their way across the bridge [117]. In this study, Cattin et al. [117] found that macrophages respond to hypoxia within the bridge by secreting VEGF-A, which triggers the polarized formation of new blood vessels across the bridge region. These new blood vessels are then used by SCs as a path to cross the bridge and guide the regrowing axons. The authors show that these newly formed blood vessels are critical for SC guidance. Indeed, in vivo disruption of their organization either by angiogenic signal inhibition or by forcing their re-orientation compromises SC directionality, resulting in defective nerve repair [117].

### STAT3 and repair SCs

STAT3 has been identified as a critical factor promoting a regeneration-supportive environment following nerve injury. Indeed, Benito et al. [118] showed that STAT3 activation by phosphorylation of Tyr705 in SCs is sustained during long-term denervation and is required for both the maintenance of SC autocrine survival signals and the maintenance of the SC repair phenotype during the regeneration process. In this study, the authors show that STAT3 ablation results in an abnormal morphology of both repair cells and regeneration tracks, failure to sustain the expression of key markers of repair SCs such as c-Jun, Olig1 and Shh, and reduction in the expression of regeneration supportive factors including GDNF and BDNF in the distal stumps [118].

### Notch signaling

The Notch signaling has been identified as a complex regulatory pathway which plays important functions in SCs, both during development and adulthood [119, 120]. Indeed, Notch promotes the generation of SCs from SC precursors and regulates SC proliferation in the developing stage [119]. Notch inhibits myelination and its expression is downregulated at the beginning of the myelination process [121]. Moreover, Notch antagonistic activity to Krox20 classifies it as a negative regulator of myelination [17]. In adult nerves, Notch dysregulation results in demyelination, which suggests an involvement in the signaling pathway that induces myelin breakdown in vivo. Indeed, inhibition of Notch signaling in adult mice decelerates myelin breakdown that occurs after a nerve lesion [120]. In addition, Wang et al. [122] showed how the addition of Jagged1, a Notch activator, in rat injured nerves enhances functional nerve repair, suggesting that Notch stimulation in SCs could represent an interesting therapeutic strategy promoting nerve repair.

## Conclusion

The PNS displays a remarkable ability to regenerate following injury. This process involves the coordinated action of multiple cell types and signaling pathways. Early after injury, damaged axons respond by generating a distress signal detected by SCs, which initiate the repair program. SCs respond by undergoing dynamic reprogramming and assuming an alternative differentiation state suited to meet the specific requirements arising from the injured condition. Although peripheral nerves display an impressive regenerative capacity as compared to the CNS, recovery for patients suffering from traumatic injuries and others peripheral neuropathies is often incomplete. This is mainly a result of the slow regeneration rate, which can reach approximately 1 mm per day, depending on the lesion site, and on the absence of a long-lasting repair-supportive environment. Moreover, the PNS repair ability decreases over time. SCs slowly lose their plasticity in an age-dependent way and the PNS environment becomes unsupportive to regeneration [45, 123]. Therefore, a greater understanding of the mechanisms driving SC plasticity is of utmost interest. Extensive research has been devoted to highlight the molecular mechanisms involved in SC reprogramming to provide mechanistic insights and novel therapeutic strategies to treat peripheral nerve injuries as well as to identify possible correlations with CNS mechanisms and strategies to improve CNS regeneration. Recent work demonstrates the involvement of morphogenetic transformations, epigenetic mechanisms and highlight many transcription factors and signaling pathways critical in this process. However, their induction and the temporal/quantitative activation as well as their mutual interactions have not been completely elucidated and future work should focus on learning how to manipulate repair cells, how to increase their repair-supportive functions, and how to extend their actions to meet the long periods required for axonal regeneration in clinical environment.
